# Pulmonary Hypertension as a Predictor of Early Outcomes of Mitral Valve Replacement: A Study in Rheumatic Heart Disease Patients

**DOI:** 10.7759/cureus.20070

**Published:** 2021-12-01

**Authors:** Omer Farooq, Azam Jan, Usman Ghani, Usman Qazi, Waasay Hassan Khan, Sundus Alam, Muhammad Junaid Khan, Omair A Khan, Nabil I Awan, Hussain Shah

**Affiliations:** 1 Cardiothoracic Surgery, Rehman Medical Institute, Peshawar, PAK; 2 Surgery, Hayatabad Medical Complex Peshawar, Peshawar, PAK; 3 Internal Medicine, Khushal Medical Center, Peshawar, PAK; 4 Medicine, Rehman Medical Institute, Peshawar, PAK; 5 Orthopaedic Surgery, Hayatabad Medical Complex Peshawar, Peshawar, PAK; 6 Internal Medicine, Hayatabad Medical Complex Peshawar, Peshawar, PAK

**Keywords:** mvr, severe ph, rheumatic heart disease, mitral valve replacement, pulmonary hypertension

## Abstract

Aim

Mitral valve pathology in rheumatic heart disease patients is a common cause of secondary pulmonary hypertension (PH). Our aim was to evaluate pulmonary hypertension severity as a predictor of in-hospital mortality and early complications following mitral valve replacement.

Methods

A retrospective review of rheumatic heart disease patients who underwent mitral valve replacement between January 2017 and August 2020 was performed. Systolic pulmonary artery pressure (sPAP) was used to classify patients as no PH (<35 mmHg), mild PH (35-44 mmHg), moderate PH (45-59 mmHg) or severe PH (>60 mmHg). Patients subjected to additional cardiac procedures (such as aortic valve replacement and coronary artery bypass grafting) were excluded from the study sample.

Results

The study group was composed of 159 patients (mean age: 40; 73 male, 86 female) categorized as no PH (n = 32; 20.1%), mild PH (n = 14; 8.8%), moderate PH (n = 65, 40.9%) and severe PH (n = 48, 30.2%) groups. Patient demographic data and preoperative comorbidities were comparable among the four groups. Use of intraoperative and postoperative blood products was similar in all the groups. Severe PH patients had similar in-hospital mortality (4.2%; p = 0.74) as in groups with lesser degrees of pulmonary hypertension. Likewise, increasing severity of pulmonary hypertension did not confer any significant increase in early postoperative complications, namely prolonged ICU stay (10.4%; p = 0.41), prolonged ventilation (2.1%; p = 0.70), reintubation (4.2%; p = 0.90), reopening for bleeding tamponade (6.3%; p = 0.39), new-onset renal failure (6.3%; p = 0.91), postoperative stroke (4.2%; p = 0.52) or prolonged length of stay (mean: 5.6 + 2.8 days; p = 0.49).

Conclusions

Increasing severity of pulmonary hypertension does not appear to have a significant impact on in-hospital mortality or early postoperative outcomes of patients undergoing mitral valve replacement.

## Introduction

Rheumatic heart disease is a substantial burden in developing countries where it is responsible for significant cardiovascular morbidity and mortality [1]. The leading cardiovascular consequence is mitral valve pathology, with pulmonary hypertension frequently observed secondary to left-sided valve disease [2]. The mechanism is retrograde transmission of the increased left atrial pressure, which in turn leads to pulmonary vascular remodeling and subsequently pulmonary hypertension [3]. Globally, the prevalence of pulmonary hypertension is 1%; almost 80% of those affected reside in developing countries [4].

The development of pulmonary hypertension in mitral valve disease indicates poor overall prognosis and is therefore considered an indication for early surgical intervention [3]. Although increasing severity of pulmonary hypertension in mitral valve disease patients may be an indicator of advanced disease and thus poor long-term outcomes [5], the effect of pulmonary hypertension severity on early surgical outcomes of mitral valve replacement remains an interesting debate. In the 1970s, the operative mortality of surgery for mitral stenosis with severe pulmonary hypertension (PH) was as high as 30% [6,7]. Even though recent studies have shown more favorable mortality rates ranging from 6% to 12%, severe PH remains a concern for cardiac surgeons evaluating patients for mitral valve surgery [8-11].

The aim of this study was to identify whether systolic pulmonary artery pressure (sPAP), measured via preoperative echocardiography, could serve as a useful predictor of in-hospital risk in patients undergoing mitral valve replacement surgery. Our study focused exclusively on rheumatic heart disease patients.

## Materials and methods

Patient selection

A retrospective observational study of 159 rheumatic heart disease patients who underwent mitral valve replacement during the period of January 2017 to August 2020 was conducted. Limiting the study target population exclusively to rheumatic heart disease patients provided a homogenous sample with matching preoperative variables among all groups. All patients in the study sample underwent mitral valve surgery under guidelines recommended by American Heart Association. The exceeding majority of patients received mechanical valves (n = 155), with the remaining few receiving bioprosthetic valves. Researchers used the hospital’s digital software to extract detailed patient data, which were subsequently verified and supplemented with physical files kept in the hospital records room. In order to limit biased outcomes, patients who underwent other cardiac procedures (such as aortic valve replacement and coronary artery bypass grafting) were excluded from the study sample. The study was authorized by the hospital research ethics committee. Statistical analysis, including Chi Square tests, analysis of variance (ANOVA) and independent t-tests, was performed using Statistical Package for the Social Sciences (SPSS; IBM Corp., Armonk, New York).

Pulmonary pressure assessment

Preoperative echocardiography was used to estimate pulmonary artery pressure in all patients. Right ventricular systolic pressure (RVSP) measured via echocardiography is a direct estimate of the systolic pulmonary artery pressure (sPAP) in the absence of any right ventricular outflow tract obstruction [12].

Pulmonary hypertension classification based on sPAP values is as follows: mild, 35-44 mmHg; moderate, 45-59 mmHg; and severe, >60 mmHg [13]. Based on these cutoffs, the patients in this study were classified into one of four groups: no PH, mild PH, moderate PH or severe PH.

## Results

The study population comprised 73 male (45.9%) and 86 female (54.1%) patients. The mean age of the sample population was 40.47 years (range: 12-70) with a standard deviation of 11.9. The classification of patients on the basis of severity of pulmonary hypertension was based on sPAP as follows: mild = 35-44 mmHg; moderate = 45-59 mmHg; and severe ≥ 60 mmHg [13]. Based on these cutoffs, 32 patients had no PH, 14 had mild PH, 65 had moderate PH and 48 had severe PH.

Preoperative characteristics, chronic comorbidities and medications are shown in Table 1.

**Table 1:** Preoperative characteristics, comorbidities and medications PH: pulmonary hypertension, SD: standard deviation, CAD: coronary artery disease, CVA: cerebrovascular accident, MI: myocardial infarction, NYHA: New York Heart Association, MR: mitral regurgitation, TR: tricuspid regurgitation, ACE: angiotensin-converting enzyme.

	No PH (n = 32)	Mild PH (n = 14)	Moderate PH (n = 65)	Severe PH (n = 48)
Age, yr, mean + SD	37.5 + 10.9	39.3 + 13.9	42.2 + 12.4	40.6 + 11.1
Female sex, n (%)	16 (50)	9 (64.3)	38 (58.5)	23 (47.9)
Diabetes, n (%)	4 (12.5)	3 (21.4)	10 (15.4)	4 (8.3)
Hypertension, n (%)	11 (34.4)	5 (35.7)	19 (29.2)	12 (25)
CAD, n (%)	2 (6.3)	2 (14.3)	4 (6.2)	1 (2.1)
Tobacco use, n (%)	0	2 (14.3)	3 (4.6)	2 (4.2)
Chronic lung disease, n (%)	0	0	2 (3.1)	2 (4.2)
CVA, n (%)	1 (3.1)	0	7 (10.8)	0
Renal disease, n (%)	0	1 (7.1)	4 (6.2)	3 (6.3)
Previous MI, n (%)	0	1 (7.1)	3 (4.6)	3 (6.3)
NYHA III/IV, n (%)	14 (43.8)	3 (21.4)	35 (53.8)	29 (60.4)
Atrial fibrillation, n (%)	1 (3.1)	1 (7.1)	4 (6.2)	6 (12.5)
Severe MR, n (%)	14 (43.8)	7 (50)	28 (43.1)	18 (37.5)
Severe TR, n (%)	5 (15.6)	2 (14.3)	5 (7.7)	11 (22.9)
Concomitant tricuspid repair, n (%)	3 (9.4%)	3 (21.4%)	8 (12.3%)	8 (16.7%)
Previous cardiac surgery, n (%)	1 (3.1)	0	3 (4.6)	3 (6.3)
Perfusion time, min, mean + SD	86.3 + 26.5	95.6 + 35.1	87 + 36.6	94.2 + 28.4
Cross clamp time, min, mean + SD	59.3 + 23.6	64.2 + 30.5	55 + 21.7	59.6 + 20.6
Medications				
Beta blockers, n (%)	13 (40.6)	5 (35.7)	27 (41.5)	21 (43.8)
ACE inhibitors, n (%)	6 (18.8)	1 (7.1)	3 (4.6)	6 (12.5)
Nitrates, n (%)	2 (6.3)	2 (14.3)	9 (13.8)	9 (18.8)
Warfarin, n (%)	8 (25)	3 (21.4)	7 (10.8)	13 (27.1)
Aspirin, n (%)	10 (31.3)	6 (42.9)	20 (30.8)	19 (39.6)
Statins, n (%)	5 (15.6)	1 (7.1)	8 (12.3)	4 (8.3)
Ejection fraction, n (%)				
<40	0	0	2 (3.1)	1 (2.1)
40-55	9 (28.1)	5 (35.7)	18 (27.7)	13 (27.1)
>55	23 (71.9)	9 (64.3)	45 (69.2)	34 (70.8)

**Figure 1:**
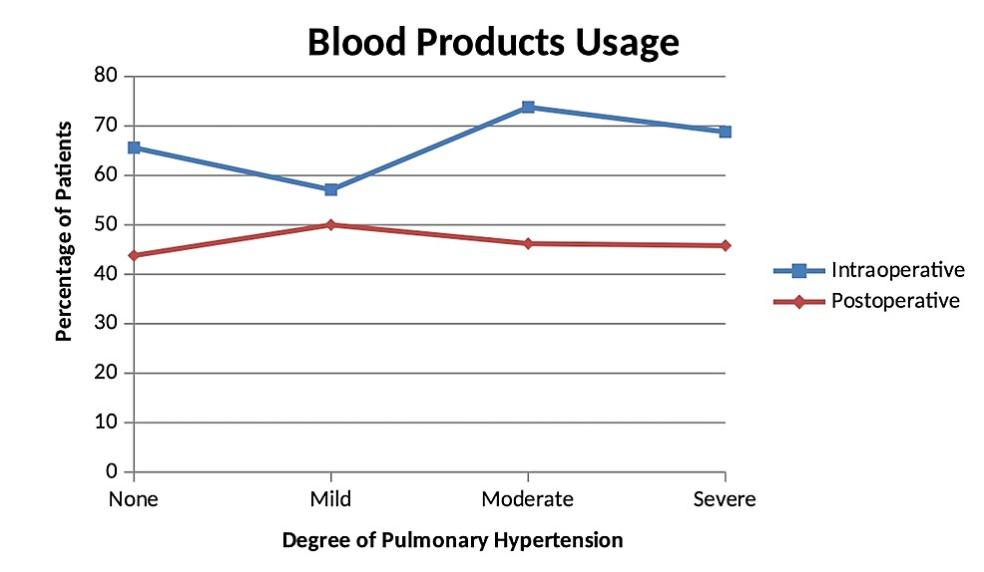
Blood products usage

Blood products usage, both intraoperative and postoperative, is shown among all the groups in Figure 1. Blood products refer to any of the following: red blood cells, fresh frozen plasma, cryoprecipitate, platelets or whole blood. Intraoperative blood products usage (p = 0.62) was as follows: no PH = 21 (65.6%); mild PH = 8 (57.1%); moderate PH = 48 (73.8%); and severe PH = 33 (68.8%). Postoperative blood products (p = 0.98) were used as follows: no PH = 14 (43.8%); mild PH = 7 (50%); moderate PH = 30 (46.2%); and severe PH = 22 (45.8%). Ultimately, there was no significant difference in blood products usage, either intraoperative or postoperative, with increasing severity of pulmonary hypertension.

Table 2 shows in-hospital mortality and early complications in patients of all four groups. In-hospital mortality comprises patients who were declared dead during the same hospitalization. New-onset renal failure requiring dialysis, as shown in Table 2, refers to a new requirement for dialysis or an increase of serum creatinine to more than 2.0 mg/dL and double the most recent preoperative creatinine level. Other rare complications that were not mentioned in the table include 1) pleural effusion in three patients in the severe PH group, 2) deep sternal infection in one patient in the moderate PH group, 3) ventricular tachycardia in one patient in the severe PH group and 4) heart block in one patient in the mild PH group.

**Table 2:** In-hospital mortality and early complications PH: pulmonary hypertension, ICU: intensive care unit, SD: standard deviation.

	No PH (n = 32)	Mild PH (n = 14)	Moderate PH (n = 65)	Severe PH (n = 48)	p-value
In-hospital mortality, n (%)	1 (3.1)	0	4 (6.2)	2 (4.2)	0.74
Prolonged ICU stay (>48 hours), n (%)	5 (15.6)	3 (21.4)	5 (7.7)	5 (10.4)	0.41
Initial hours ventilated postoperatively, hrs, mean + SD	4.8 + 4.7	5.8 + 7.2	5.9 + 8.1	6.7 + 7.8	0.73
Prolonged ventilation (>24 hours), n (%)	0	0	2 (3.1)	1 (2.1)	0.7
Reintubated during hospital stay, n (%)	1 (3.1)	1 (7.1)	2 (3.1)	2 (4.2)	0.9
Reopening for bleeding tamponade, n (%)	0	0	4 (6.2)	3 (6.3)	0.39
New-onset renal failure requiring dialysis, n (%)	1 (3.1)	1 (7.1)	4 (6.2)	3 (6.3)	0.91
Cerebrovascular accident within 72 hours, n (%)	0	0	1 (1.5)	2 (4.2)	0.52
Length of stay, days, mean + SD	5.7 + 5.9	4.6 + 0.6	4.9 + 1.4	5.6 + 2.8	0.49

## Discussion

Pulmonary hypertension is a common finding in patients with left-sided valve pathology, for instance, in patients with long-standing rheumatic heart disease. The major pathomechanism is retrograde transmission of pressure from the left atrium due to poor forward flow; however, active resistance resulting from pulmonary vascular vasoconstriction and remodeling may also contribute to pulmonary hypertension in long-standing left heart disease. Gradually, patients also develop right ventricular dysfunction and dilation [14,15]. Although early intervention can improve hemodynamic parameters following correction of left heart pathology, severe chronic PH may result in irreversible changes. This is especially true for patients in developing countries where delayed presentation is a common phenomenon. Seventy-eight percent of patients undergoing mitral valve surgery are reported to have concomitant pulmonary hypertension [16]. Given the abundance of cases of mitral valve disease presenting with severe pulmonary hypertension, we sought to evaluate severe pulmonary hypertension as an independent predictor of in-hospital risk following mitral valve surgery.

Severe PH has been, for a long period of time, a major concern of surgeons operating mitral valve disease, with earlier studies reporting mortality up to 30% in these patients [6,9]. Najafi et al. found that perioperative mortality strongly correlates with the degree of pulmonary hypertension, ranging from 16% in patients with mild pulmonary hypertension to 61% in patients with high pulmonary pressures equivalent of systemic pressures [7]. However, the numbers have substantially improved in more recent studies. Mubeen et al. reported that mitral valve surgery is not associated with significant mortality even in severe pulmonary hypertension as long as pulmonary pressures remain below systemic pressures [10]. Despite the conflicting data reported in the literature, severe PH remains a concern in patients considered for mitral valve surgery.

When evaluating outcomes of mitral valve surgery in patients with pulmonary hypertension, one particularly important aspect to address is the effect of concomitant cardiac procedures. Vincens et al. reported greater mortality rates in their study compared to others, potentially due to inclusion of patients requiring coronary artery bypass grafting (CABG) (37% of the total number of patients) as well as aortic valve replacement (42% of the total patients) [11]. On the other hand, McIlduff et al. reported a relatively lower mortality rate of 7.7% with lower concomitant CABG (15%) and aortic valve replacement (27%) [8]. To reduce the effect of these confounding factors, we excluded patients requiring CABG and aortic valve replacement from our study.

Our study showed no increase in perioperative morbidity or mortality in patients with severe pulmonary hypertension as compared to those with no, mild or moderate pulmonary hypertension. This is in accordance with recent studies [10] and in contrast to studies done decades earlier [6] which reported significantly higher mortality rates around 50%. This may be due to improved surgical techniques being developed as well as the advanced medical technology employed in hospitals nowadays. Furthermore, in our study, the severity of pulmonary hypertension did not correlate significantly with early (30 days) postoperative complications.

In summary, the results of our study revealed that mitral valve surgery can be performed with acceptable in-hospital mortality and early complication rates in patients with varying degrees of pulmonary hypertension. However, other factors like long-term survival, concomitant heart disease and valvular pathology also play a major role in selecting patients for mitral valve replacement and need to be taken into consideration.

## Conclusions

Our retrospective analysis of rheumatic heart disease patients revealed that severity of pulmonary hypertension does not reliably correlate with in-hospital mortality or early complications in patients undergoing mitral valve surgery. Although higher degrees of pulmonary hypertension may correlate with advanced disease, patients undergoing mitral valve replacement have favorable early outcomes regardless of the severity of pulmonary hypertension.
